# SpolPred: rapid and accurate prediction of *Mycobacterium tuberculosis* spoligotypes from short genomic sequences

**DOI:** 10.1093/bioinformatics/bts544

**Published:** 2012-09-26

**Authors:** Francesc Coll, Kim Mallard, Mark D. Preston, Stephen Bentley, Julian Parkhill, Ruth McNerney, Nigel Martin, Taane G. Clark

**Affiliations:** ^1^Faculty of Infectious and Tropical Diseases, London School of Hygiene and Tropical Medicine, London, ^2^Wellcome Trust Sanger Institute, Wellcome Trust Genome Campus, Hinxton and ^3^Department of Computer Science and Information Systems, Pathogen Genomics, Wellcome Trust Sanger Institute, Wellcome Trust Genome Campus, Birkbeck College, London, UK

## Abstract

**Summary**: Spoligotyping is a well-established genotyping technique based on the presence of unique DNA sequences in *Mycobacterium tuberculosis* (*Mtb*), the causal agent of tuberculosis disease (TB). Although advances in sequencing technologies are leading to whole-genome bacterial characterization, tens of thousands of isolates have been spoligotyped, giving a global view of *Mtb* strain diversity. To bridge the gap, we have developed *SpolPred*, a software to predict the spoligotype from raw sequence reads. Our approach is compared with experimentally and *de novo* assembly determined strain types in a set of 44 *Mtb* isolates. *In silico* and experimental results are identical for almost all isolates (39/44). However, *SpolPred* detected five experimentally false spoligotypes and was more accurate and faster than the assembling strategy. Application of *SpolPred* to an additional seven isolates with no laboratory data led to types that clustered with identical experimental types in a phylogenetic analysis using single-nucleotide polymorphisms. Our results demonstrate the usefulness of the tool and its role in revealing experimental limitations.

**Availability and implementation**: *SpolPred* is written in C and is available from www.pathogenseq.org/spolpred.

**Contact:** francesc.coll@lshtm.ac.uk

**Supplementary information:** Supplementary data are available at *Bioinformatics* Online.

## 1 INTRODUCTION

Tuberculosis is an infectious disease caused by bacterium of the *Mycobacterium tuberculosis* (*Mtb*) complex. Genotyping techniques based on the presence of repetitive elements, conserved sequences, or loci with variable numbers of tandem repeats have been standardized (Kanduma *et al.*, 2003), allowing the comparison of isolates between laboratories and regions worldwide. The popular spoligotyping approach (Kamerbeek *et al.*, 1997) exploits the polymorphism at the direct repeat (DR) locus of *Mtb*. It is based on the polymerase chain reaction (PCR) amplification of 43 short unique sequences (termed spacers) found between well-conserved 36-bp DRs and the subsequent hybridization of the products onto a membrane with oligonucleotides complementary to each spacer. Since strains vary in the occurrence of particular spacers, each sample produces a distinctive spot pattern, which is then translated into a numerical code of 15 digits known as octal code (Dale *et al.*, 2001). The web-based database SITVITWEB contains 2740 shared types or spoligotype international types (SITs) found among 58 180 clinical isolates, subsequently grouped into a list of 62 lineages/sublineages which normally show a geographic distribution (Demay *et al.*, 2012). *In silico* genotyping approaches are required to bridge the gap between experimental and high-throughput sequencing, leading to the development of *SpolPred*, a software to predict the spoligotype from raw sequence reads.

## 2 METHODS

We have developed a C executable to predict the spoligotype octal code from files of *fastq* format. By making use of a 2-bit per nucleotide coding strategy to speed up performance, every 25-bp unique spacer is queried against each read allowing up to one mismatch (Ioerger *et al.*, 2009). The spacer sequences chosen are the same as those used as probes in the original spoligotyping assay (Kamerbeek *et al.*, 1997). The read length can be changed to support data from different (single or paired end, minimum 35 bp) sequencing platforms (see User Manual for more available options). The appearance of all 43 queries is eventually translated into the octal code which is then matched to a spoligotype in SITVITWEB. The software has been applied to 51 Ugandan *Mtb* isolates which underwent sequencing using Illumina–GAII 76-bp paired end technology at the Sanger Institute. The number of paired reads varied per sample from 10 to 30 million. To evaluate accuracy, *Spolpred*-predicted results were compared with those experimentally determined for 44 samples in our laboratory. In addition, results were compared with those manually extracted from *de novo* genome assemblies, generated using *Velvet* (Zerbino and Birney, 2008) [default settings, except 51k-mer word length, insert length (300 bp) and its standard deviation (30 bp)]. Subsequently, the same 25-bp spacers were queried against the resulting contigs. A cluster dendogram was constructed using 6998 single-nucleotide polymorphisms (SNPs) to allow the investigation of seven isolates with no experimental data.

## 3 RESULTS

**Fig. 1.**
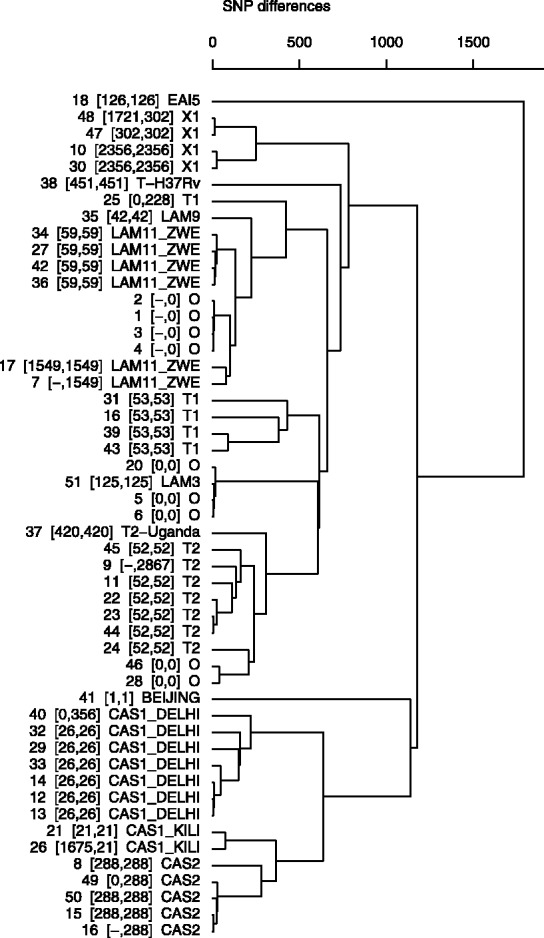
Dendogram for 51 Ugandan isolates constructed using 6998 SNPs, showing (from left to right): isolate number (experimentally, *SpolPred* determined SIT code) and *SpolPred* inferred spoligotype;– indicates no laboratory data available

The software was tested on a 64-bit Ubuntu Linux computer with a 3.07 GHz processor and 8 GB of RAM. As expected, running time per *fastq* file increased proportionally with read coverage. Nevertheless, processed reads per unit of time remained constant (~500 000 reads per minute). *SpolPred*-inferred SIT numbers matched the experimental ones for 39 samples (88.6%). The resulting octal codes from both *in silico* approaches were identical for 42 (95.5%) of the 44 samples with experimental results (see Supplementary Table S1 for detailed results). In the remaining two isolates, *de novo* assembly failed to detect spacer 25, which happens to fall between contiguous assembled contigs. The overall five non-matched *in silico* and experiment results were due to the increased *in silico* sensitivity of the detection of spacer 15 in the five samples and, additionally, spacer 26 in one sample. When the original hybridization blots were checked, an irregular signal distribution for spacer 15 across all samples was noted. Some signals were either too faint or just not detectable to be manually assigned as being present. Although predicted spoligotypes remained unchanged for samples 26 and 48, the other three (25, 40 and 49), which had octal codes not previously reported in the SITVITWEB database, were re-assigned to different spoligotypes. Interestingly, these three isolates are consistently clustered in the SNP-based dendogram, i.e. within a clade of samples having the same experimental type. Similarly, all samples with no laboratory data were clustered with isolates of the same predicted spoligotype (Fig. 1).

## 4 DISCUSSION

Although SNPs and other genetic variation derived from sequencing projects are likely to become the markers of choice due to their discriminatory power, PCR genotyping techniques are still widely employed. In this regard, *SpolPred* will enable the complementary comparison of computationally inferred spoligotypes with laboratory results. Currently, the tedious parameter optimization and computational requirements for *de novo* assembly are important constraints. Only 42 (95.5%) spoligotypes could be accurately inferred using the *de novo* strategy implemented. The region in the DR locus harbouring spacer 25 does not seem to be reconstructed in two genomes, resulting in an incorrect type classification. Importantly, in those five samples for which *SpolPred* and experimental patterns differ, predicted octal codes were exactly the same when using both computational approaches. Furthermore, the newly assigned spoligotypes (namely, isolates 25, 40 and 49 in Fig. 1) are clustered with other isolates having coincident experimental and *in silico* predicted lineages. The absent sequence responsible for the discrepancies observed, namely, spacer 15, was the same across all five problematic isolates. The ambiguous distinction of this spacer has been reported (Abadia *et al.*, 2011) and explained in terms of the presence of a 4-nt deletion adjacent to the amplified sequence (van Embden *et al.*, 2000), which would not allow a proper primer hybridization. Other ambiguities caused by the insertion of IS6110 copies in the DR region have also been reported (Filliol *et al.*, 2000). As demonstrated, the software can be employed to accurately and quickly confirm experimentally determined spoligotypes, infer them from sequenced isolates with no laboratory data and reveal unexpected cases of wrongly assigned types. Other causes of TB misclassification such as laboratory cross contamination, PCR contamination or ambiguous hybridization patterns could also be clarified. With the amount of sequence data increasingly growing, software like *SpolPred* will be useful additions to pipelines used to infer TB (e.g. MIRU-VNTR) and other bacterial strain types (e.g. MLST typing) and ultimately assist with disease control.

*Funding*: This work was supported by a Bloomsbury Colleges PhD Studentship. The Wellcome Trust supports the Sanger Institute.

*Conflict of Interest*: none declared.

## Supplementary Material

Supplementary Data
