# Supplementary material for: SpolPred: rapid and accurate prediction of Mycobacterium tuberculosis spoligotypes from short genomic sequences
Source: Bioinformatics. 2012 Sep 26;28(22):2991–3. doi: 10.1093/bioinformatics/bts544 (PMC3496340; doi:10.1093/bioinformatics/bts544)
Supplement: Supplementary Data [file supp_28_22_2991__index.html]

SpolPred: Rapid and accurate prediction of Mycobacterium tuberculosis spoligotypes from short genomic sequences — SpolPred: rapid and accurate prediction of Mycobacterium tuberculosis spoligotypes from short genomic sequences — Supplementary Data 

# SpolPred: rapid and accurate prediction of *Mycobacterium tuberculosis* spoligotypes from short genomic sequences

## Supplementary Data

files

**Files in this Data Supplement:**

- Supplementary Data - xlsx file
- Supplementary Data - docx file
