# Supplementary material for: SpolPred: rapid and accurate prediction of Mycobacterium tuberculosis spoligotypes from short genomic sequences
Source: Bioinformatics. 2012 Sep 26;28(22):2991–3. doi: 10.1093/bioinformatics/bts544 (PMC3496340; doi:10.1093/bioinformatics/bts544)
Supplement: Supplementary Data [file supp_bts544_UserManual_SpolPred.docx]

SPOLPRED USER MANUAL

1. **Installation**

SpolPred can be compiled on any Linux environment with g++, the C++ compiler.

> g++ spolpred

> mv a.out spolpred

Now the executable file named *spolpred* can be run. If not installed, open a terminal window and type:

> sudo apt-get install build-essential

1. **Running instructions**

**2.1 Command line**

./SpolPred <reads.fastq> [-l <ReadLength>] [-b <d/r>] [-o <output.txt>] [-d <on/off>] [-s <on/off>] [-a <ScreeningThreshold>] [-m <MatchingThreshold>]

**2.2 Input sequence file**

SpolPred accepts one sequence file in FASTQ format. Both direct and reverse reads are supported.

**2.3 Options**

|  | Default Value | Description |
| --- | --- | --- |
|  |  |  |
| -l <ReadLength> | 75 | Read length. Minimum 35 bp. |
|  |  |  |
| -b <d/r> | d | Direct (d) or reverse (r) input reads. |
|  |  |  |
| -o <output.txt> | output.txt | Output file name. |
|  |  |  |
| -d <on/off> | on | If set on, processing details are output on the terminal screen including number of processed reads and number of spacer sequences found. |
|  |  |  |
| -s <on/off> | off | Stop Screening option. Used to end read processing when Screening Threshold is reached |
|  |  |  |
| -a <ScreeningThreshold> | 50 | Average number of spacer occurrences used to stop screening. |
|  |  |  |
| -m <MatchingThreshold> | 4 | Minimum number of spacer occurrences below which spacer absence is assigned. |
|  |  |  |

**2.4 Output**

The text file output is expected to look like follows:

sample.fastq 700377740003771 113 135 108 0 0 1 0 0 0 0 171 132 116 144 115 139 121 122 117 59 55 112 0 0 0 1 0 0 0 0 0 0 0 0 15 0 144 93 111 108 164 136 138 118 135 109 0 0 1 0 0 0 0 208 133 116 149 117 142 123 123 122 60 56 114 0 0 0 1 0 0 0 0 0 0 0 0 15 24 149 106 112 108 166 140 140

Sample name, octal code, read-spacer exact matches and read-spacer matches allowing up to one mismatch are printed separated by tabs.

If the same output file name is specified in following runs, the new lines will be appended to the file

1. **Frequently asked questions.**

**SpolPred only accepts one FASTQ file, what if I have got paired-end reads?**

Forward and reverse read files can be merged into one by making use of the Perl script *shuffleSequences_fastq.pl* provided in *Velvet* software suite. SpolPred run will therefore take longer than using only forward or reverse reads. In our dataset (read Methods for more details), the forward file had enough reads to find all present spacers and infer the octal code for 49 out of 51 samples. That decision will have to be made depending on the sample coverage depth.

**What if I have a FASTA file?**

SpolPred has been particularly designed to process raw reads and therefore only supports sequence files in FASTQ format.

**What is the point of stopping the read screening?**

By default, all reads in the FASTQ file will be processed. Nevertheless, we have observed that a point is reached when no more reads are needed to infer the octal code, in other words, the number of spacer occurrences is high enough and steady to assume that all present spacers have already been found. Therefore, stopping the program at this point would save time and computer resources. If low coverage is the case, stopping the scanning is not advisable.

**How do I choose the Screening threshold?**

If you have decided to scan the whole input file there is no need to set such threshold. The Screening threshold is used to let the program know when the screening should stop. Such value will depend on read coverage. Running the software and looking at the number of times all spacers are detected will provide insight into both the coverage and the most appropriate threshold value.

**Why a Matching threshold is required, are not spacers supposed to occur uniquely?**

The number of times each spacer is found is tracked during the screening and absence assigned when such number does not reach a user-defined threshold (4 times by default). This threshold, here called Matching threshold, has had to be implemented because for some absent spacers, a few spurious matches were found. Those false positives are likely to be related with bad-quality issues, like sequencing errors. In our data set, no more than 3 false matches were detected for absent spacers, in contrast to 50-150 found per present spacer.

**Should I be worried then about false positive matches?**

As long as proper pre-filtering steps are carried out to the raw reads, no important issues are expected to come up.

**Can I change the number of allowed SNPs when querying the spacers?**

This option has not been implemented. Spacer sequences are conserved and only one SNP has been reported to occur at the most.

**Why are exact matches output as well?**

The number of read-spacer exact matches, i.e. without allowing SNPs, will enable the easily identification of SNPs on spacer sequences. When inferring the octal code, exact matches are not employed.
